# Neurasthenia: A Distinct and Specific Pathological Condition

**Published:** 1916-09-16

**Authors:** 


### NEURASTHENIA.

### A Distinct and Specific Pathological Condition.

"It is true," said Tennyson a good many years ago, "it is true that we have a faithful ally, but only the devil knows what he means." If Tennyson's life had been so prolonged that he had become acquainted with the term "neurasthenia" he might have adapted his own estimate of Louis Napoleon, and said, "It is true that we have a beautiful term, but only the devil knows what it means." In fact, even the devil himself cannot be sure of the meaning, for the word means now this, and now that, and now the other, according to the necessity of the user. In the mouths of practitioners of medicine it has all the convenient vagueness and speculativeness of a liver out of order, and in the ears of the patient and his friends it is equally satisfying and consoling. "What is the matter with my daughter, doctor, she seems very strange?" "A slight attack of neurasthenia, madam; I will send in a nurse and a bottle of medicine, and she will soon be better." So the nurse arrives, and finds the patient huddled up in a corner of the room, seated on the floor, clad in a night-gown or less, and moaning that she has committed the unpardonable sin, and that God has forgotten her. The nurse, experienced in all kinds of medical and surgical nursing, discovers that here is a case new to her experience, and promptly administers a dose of medicine as instructed, and a cup of tea on her own initiative; and is considerably surprised when the patient throws the tea in her face, and the cup and saucer out of the window. That is one kind of neurasthenia.

"He seems very bad, doctor; what do you think is the matter with him?" "Your husband is suffering from neurasthenia. He will want two nurses, one for day and one for night; I will send them in, and meantime get this prescription made up." "But, doctor, what are we to do when he tries to kill the rats and snakes, and sees devils dancing all round him? Is it not very serious?" "Common symptoms in neurasthenia, my dear madam; feed him well, and they will subside in a few days."

"Doctor, you know I have not been able to go to Homburg for two seasons. I am afraid my liver is badly out of order. My appetite is not what it should be; I was obliged to pass two courses at dinner last night, and I didn't care for the sweet at lunch, and had to make up by a little extra Stilton. I have tried carriage exercise, but it doesn't seem to give me any appetite; and I have a headache, and a feeling of stuffiness, and a devil of a pain in my great toe. You don't think it can be gout, do you? If so, it cannot be what I eat; it must be poor man's gout." "Gout? Nonsense, my dear sir; a touch of neurasthenia, that's all! Here, get this prescription made up, and as your appetite is so poor, try a boiled sole and just a small cutlet. I'll come and see you again in the morning."

"Doctor, I don't know what has come to my husband; he says he can stop the war, and he has written all these telegrams to King George, and the Kaiser, and the King of the Belgians, and the Czar, and lots of other people, saying he will call on them to-morrow, and they are to proclaim a truce till he arrives. And he has a gun, and says he will shoot the first person that goes into his room. What can be the matter with him?" "Well, madam, he seems from what you say to have rather a severe attack of neurasthenia. However, a bottle of medicine——"

These are cases that have, in the personal experience of the writer, been called "neurasthenia," and it is the custom to apply the same term to any case of such a nature that the patient and his friends would be shocked to hear called by its true name. As ordinarily applied to disease of men it has about as much meaning as the term "blight" applied to disease of plants. It is a verbal cloak, employed to cover up either the susceptibility of the patient or his friends, or the ignorance of his doctor. Another term, equivalent to neurasthenia, and used in the same sense and for the same purposes, is "nervous breakdown," a term as blessed and comforting as "Mesopotamia," and, when applied to disease, quite as destitute of meaning. It may cover anything from an outbreak of hysteria to an outbreak of acute mania; from a fit of the sulks to a fit of apoplexy.

In fact, neurasthenia is not the same as acute mania, or delirium tremens, or the results of over-eating. It is not the name of a group of diseases including these and others, as its common use would appear to indicate. It is not even a vague and convenient term like "nervous breakdown," which means nothing in particular and everything or anything in general, and may be used as a cloak for ignorance. Neurasthenia has a specific and definite meaning, and stands for a well-characterised pathological state, and he who uses it in any other sense exhibits a double measure of ignorance, and shows not only that he does not know the nature of the disease to which he applies the name, but also that he does not know the meaning of a common term. The central nervous system has, among its other functions, that of serving as a storehouse of energy. The animal body may be looked upon from one point of view as a complicated machine or group of machines all acting with various degrees of energy at various times, or resting for a time as the case may be. In a factory all the machines are supplied from a single source, the boiler in the basement, with the energy that actuates them. In the human body all the machines, the heart, the stomach, the liver, the muscles, etc., are supplied from a single source, the central nervous system, with the energy that actuates them. In the factory, if the energy from the boiler is cut off from any single machine by the severance or displacement of a belt, that machine comes to a standstill and ceases to work; it takes no part in the economy of the factory until the channel by which it receives its energy is restored. In the human body, if the energy from the central nervous system is cut off from any organ by the severance of its nerve, that organ ceases to work: it comes to a standstill, and takes no part in the economy of the body until the channel by which it receives its energy is restored. If the fire in the boiler is suffered to languish, the pressure of the steam falls and the machines work feebly, languidly, and inefficiently, and those that need the most power or are farthest from the source of supply may fail to work at all; and if the pressure of energy in the central nervous system falls, the organs of the body languish, they perform their work feebly and languidly and inefficiently, and those that need the most power, or are physiologically remote from the source of supply, may fail to act at all. This failure in the supply of energy is neurasthenia. Neurasthenia is that state of the body in which the fires of life burn low, in which little energy is generated and stored in the nervous system, in which, therefore, little is at the service of the organs of the body, and in which consequently they perform their functions feebly and languidly. The human body is so constituted that those organs which are most vitally necessary have the first call on the stored energy, and they will have their supply whatever other organs have to go without. Those that are least necessary to the maintenance of life can get only what energy is left over after the vital organs have received their supply. Hence it is that in neurasthenia the heart never fails to act until the store is completely empty, and the organs that feel the deficiency first and most are the voluntary muscles. This is why the neurasthenic is such a feeble creature, and this explains why we can find, or we may find, no lesion, no structural damage of any organ. The machines are there, sound in structure, and capable of exercising their functions to the full; but the functions fail for lack of driving power. Neurasthenia is not a common name for any disease that we do not understand or fail to diagnose, nor is it a common name for any disease that we are reluctant to call by its proper title. It is a distinct and specific pathological condition.

